# Heterogeneous nuclear ribonucleoprotein D — an understudied subfamily affected in sporadic TDP-43 proteinopathies

**DOI:** 10.1093/braincomms/fcae352

**Published:** 2024-11-01

**Authors:** Monica Pinkerton, Gabrielle L Adler, Mallory Ledger, Chen Yue Ni, Yue Yang, Rachel H Tan

**Affiliations:** Faculty of Medicine and Health, School of Medical Sciences, University of Sydney, Camperdown, Sydney, NSW, 2050, Australia; Brain and Mind Centre, University of Sydney, Sydney, NSW, 2050, Australia; Faculty of Medicine and Health, School of Medical Sciences, University of Sydney, Camperdown, Sydney, NSW, 2050, Australia; Brain and Mind Centre, University of Sydney, Sydney, NSW, 2050, Australia; Faculty of Medicine and Health, School of Medical Sciences, University of Sydney, Camperdown, Sydney, NSW, 2050, Australia; Brain and Mind Centre, University of Sydney, Sydney, NSW, 2050, Australia; Brain and Mind Centre, University of Sydney, Sydney, NSW, 2050, Australia; Faculty of Medicine and Health, School of Medical Sciences, University of Sydney, Camperdown, Sydney, NSW, 2050, Australia; Brain and Mind Centre, University of Sydney, Sydney, NSW, 2050, Australia

**Keywords:** amyotrophic lateral sclerosis, frontotemporal lobar degeneration, pTDP-43

## Abstract

Despite the recognition that heterogeneous nuclear ribonucleoproteins (hnRNPs) modulate TDP-43 and can limit aberrant splicing events to compensate for TDP-43 loss, their role in TDP-43 proteinopathies remains poorly understood and studies in patient tissue are lacking. This study assesses seven heterogeneous nuclear ribonucleoproteins from the A/B, C, D and H subfamilies in two cortical regions implicated in early TDP-43 dysfunction versus late TDP-43 dysfunction in sporadic amyotrophic lateral sclerosis and/or frontotemporal lobar degeneration. Our results reveal significant nuclear loss of hnRNPD, hnRNPC and hnRNPA1 in the frontal cortex of frontotemporal lobar degeneration compared to amyotrophic lateral sclerosis but not in the motor cortical neurons or Betz cells of amyotrophic lateral sclerosis cases. Cytoplasmic co-occurrence was observed between hnRNPA1 and hnRNPC but not with phosphorylated TDP-43 (pTDP-43). Interestingly, nuclear hnRNPD loss associated with increasing cytoplasmic pTDP-43, highlighting an understudied subfamily in sporadic TDP-43 proteinopathies. In summary, this study identifies the nuclear loss of hnRNPD, C and A1 in a predilection brain region of TDP-43 in frontotemporal lobar degeneration compared to amyotrophic lateral sclerosis cases without significant pTDP-43 in this region. This highlights the need for further investigation into the involvement of these heterogeneous nuclear ribonucleoproteins in disease pathogenesis and potential to serve as modulatory targets and/or proximal markers of TDP-43 dysfunction in sporadic TDP-43 proteinopathies.

## Teaser

Pinkerton *et al.* demonstrate significant nuclear depletion of heterogeneous nuclear ribonucleoproteins (hnRNPs) in the frontal cortex of patients with sporadic frontotemporal dementia compared to amyotrophic lateral sclerosis but not in the motor cortical neurons or Betz cells, highlighting the need for further investigation into understudied hnRNPs in sporadic TDP-43 proteinopathies.

## Graphical Abstract

**Graphical Abstract:**
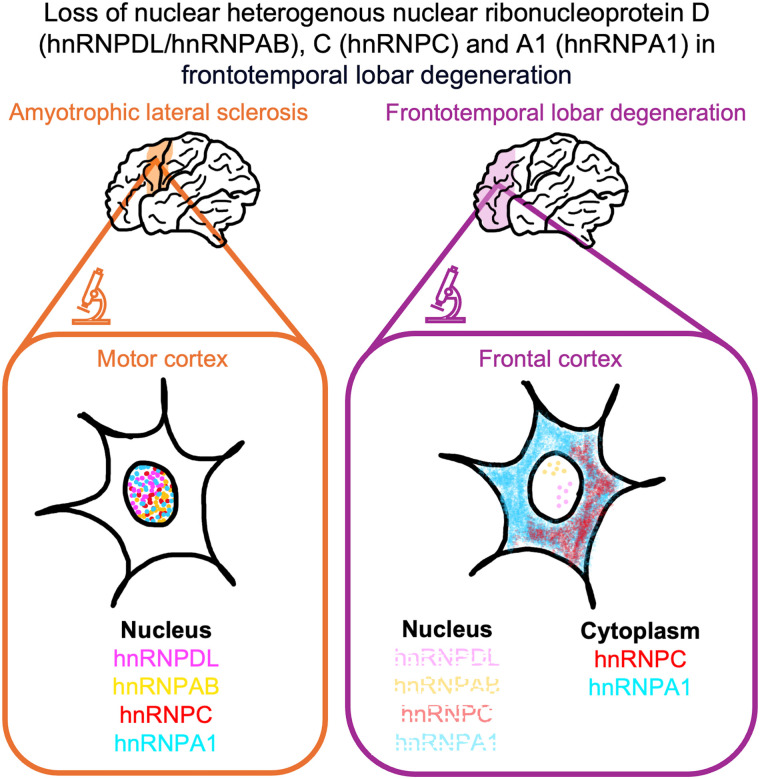

## Introduction

TDP-43 is frequently categorized as a member of the heterogeneous nuclear ribonucleoprotein (hnRNP) family due to its structural similarities and like other members of the hnRNP family, TDP-43 plays a critical role in the regulation of many important aspects of RNA processing.^1,2^ Cytoplasmic aggregates of the normally nuclear TDP-43 protein are a major pathological hallmark of amyotrophic lateral sclerosis (ALS) and frontotemporal lobar degeneration (FTLD), which are two neurodegenerative diseases believed to represent a disease continuum.^3^ Recent evidence suggests the loss of TDP-43 repression of cryptic splicing following nuclear TDP-43 depletion as a driver of neurodegeneration in ALS and FTLD.^4^ Importantly, other hnRNP proteins have been shown to interact with and modulate TDP-43 function as well as limit aberrant splicing events in the absence of TDP-43 to compensate for its loss, thereby representing promising disease modifiers of TDP-43 induced toxicity.^5,6^ The last few years has seen increasing momentum towards the development of hnRNP-targeting compounds to limit tumour progression and overcome drug resistance in various types of cancers.^7^ Although dysfunction in hnRNPs have been identified in ALS and/or FTLD, most studies have focused on FUS-related disease and few studies have sought to determine if hnRNPs are similarly affected across sporadic TDP-43 proteinopathies.^8^ As such, the present study comparatively assesses seven hnRNPs from the hnRNP A/B, C, D and H subfamilies (A1, A2B1, A0, C, DL, AB and H2) in two cortical regions implicated in early and late stages of TDP-43 pathogenesis in sporadic ALS, ALSFTLD and FTLD cases.

## Materials and methods

### Cases

Cases with a pathological diagnosis of FTLD-TDP and/or ALS-TDP were selected from a neuropathological series collected by the Sydney Brain Bank. Patients were diagnosed during life by experienced clinicians using standard clinical diagnostic criteria. Standardized neuropathological characterisation were performed and cases had previously been screened for mutations in the *C9ORF72* and *GRNs* genes.^9^ Given the specific focus on pathological TDP-43, only cases without genetic mutations and without co-existing Alzheimer’s disease and/or Lewy body disease were selected for this study. Thirty cases met these inclusion criteria and were categorized into four age- and sex-matched groups of ALS-TDP cases (*n* = 11; 55% male, mean ± SED: 65 ± 3 years at death), FTLD-TDP cases with concomitant ALS (FTLD-ALS, *n* = 6; 50% male, mean ± SED: 64 ± 2 years at death), FTLD-TDP cases characterized by cytoplasmic inclusions (FTLD-NCI, *n* = 6; 67% male, mean ± SED: 76 ± 4 years at death) and FTLD-TDP cases characterized by long dystrophic neurites (FTLD-DN, *n* = 7; 86% male, mean ± SED: 74 ± 3 years at death). As expected, FTLD-DN cases demonstrated a significantly longer disease duration compared to all other groups (mean ± SED (year): 11 ± 1 in FTLD-DN; 3 ± 0 years in ALS; 2 ± 1 years in FTLD-ALS; 5 ± 1 years in FTLD-NCI; (*F*(3,26) = 22.9, *P* < 0.001)), with no significant differences between all other groups (*P* > 0.2 for all). Although previous studies on the ALS-FTLD continuum have focused on cases with neuronal TDP-43 inclusions,^6,9^ the present study included cases characterized by dystrophic neurites at the end of their longer disease duration as a comparison group. No significant difference in post-mortem interval was identified between groups (mean ± SED (h): 19 ± 3 in ALS, 15 ± 3 in FTLD-ALS, 25 ± 5 in FTLD-NCI and 27 ± 8 in FTLD-DN; (*F*(3,26) = 0.94, *P* > 0.4)). This study has approval from the HREC of the University of Sydney and complies with the statement on human experimentation by the National Health and Medical Research Council Australia.

### Cortical regions and hnRNPs assessed

The selection of the motor and frontal cortices as the regions-of-interest in this study was based on each of these regions being the predilection sites and early cortical regions targeted by TDP-43 aggregates in either disease (motor cortex in ALS, frontal cortex in FTLD) that remains unaffected or is only implicated in later stages of the other disease (motor cortex is implicated in FTLD-TDP Stage 4; frontal cortex is implicated in ALS-TDP Stage 4.^10,11^ Furthermore, we have previously shown significantly greater pTDP burden in the frontal cortices of FTLD compared to ALS cases but no significant differences in the motor cortex across FTLD and ALS cases.^9^ Previous studies have identified aberrant hnRNPE, hnRNPK and hnRNPA3 in TDP-43 proteinopathies.^12-14^ As such, the present study focused on other hnRNPs from the A/B, C, D and H subfamilies that have not been previously quantified across the ALS-FTLD continuum. Despite its name, hnRNPAB is evolutionarily distinct from the hnRNPA/B subfamily of proteins, belonging instead to the hnRNPD subfamily, which also consists of proteins with two RNA binding domains and a glycine-rich domain.^15^

### Immunohistochemistry and quantification of hnRNPs

Ten micrometre formalin-fixed, paraffin-embedded tissue blocks for each region-of-interest (ROI) were deparaffinated and antigen retrieval was performed in 0.01 M citrate buffer at 95°C for 30 min. Sections were rinsed with distilled water and blocked with Peroxidase Block and Protein Block (Novolink™ Polymer Detection Systems, Leica) for 5 min. Sections were then incubated with primary antibodies specific to anti-hnRNPA1 (Abcam, AB232824, 1:100), anti-hnRNPA2B1 (Thermofisher, PA588180, 1:200), anti-hnRNPA0 (Sigma, HPA036569, 1:800), anti-hnRNPC (SigmaAldrich, SAB4501419–100UG, 1:500), anti-hnRNPDL (SigmaAldrich, HPA056820, 1:500), anti-hnRNPAB (Sigma, HPA046688, 1:100), anti-hnRNPH2 (SigmaAldrich, HPA001359, 1:500) or anti-phosphorylated TDP (pTDP-43) (CosmoBio, TIP-PTD-M01, 1:80 000) diluted in Tris-buffered saline overnight at 4˚C. Sections were incubated with Post Primary Block (Leica) and primary antibodies were detected using Novolink™ Polymer (Leica) and visualized using a DAB (Sigma–Aldrich) solution containing cobalt and nickel ions. All slides were counterstained with haematoxylin for quantitation of neuronal populations. Normal nuclear localisation of these hnRNPs was confirmed in a control case and this is consistent with previously published studies.^16-18^ Slides were digitally scanned using the Olympus VS-200 slide scanner and analysed using the QuPath software (v0.4) as previously described.^19^ Briefly, two strips of cortex through the entire cortical thickness from the pial surface to white matter were sampled in each cortical section and overlaid with a grid (comprised of individual frames that each measured 500 µm × 500 µm). The total number of neurons with nuclear hnRNP were counted and expressed as a percentage of the total neurons in each ROI. The percentage area occupied by cytoplasmic pTDP-43 inclusions and dystrophic neurites in two strips of cortex were quantified using QuPath as detailed previously.^20^ Betz cells were identified by their large soma at lower magnification (10×) across the layer (Supplementary Fig. 2). Given that Betz cells are only located within cortical layer V, the grey matter on each motor cortex section was delineated and the total number of Betz cells alongside the % with normal nuclear localisation of hnRNP or aberrant nuclear loss of hnRNP were quantified. Quantification was conducted by two raters blind to treatment groups, with an inter-rater agreement of <5%.

### Immunofluorescence analysis

The association between pathological pTDP-43 with hnRNPs that demonstrated cytoplasmic accumulation (hnRNPA1, hnRNPC) was assessed using double-labelled immunofluorescence on 10 μm thick sections as previously described.^21^ AlexaFluor 488-labelled anti-mouse IgG (Thermofisher 1:200) and Alexa 568-labelled anti-rabbit (Thermofisher 1:200) were used as secondary antibodies and sections were counterstained with 4′,6-diamidino-2-phenylindole DAPI (Thermofisher, 62248, 1 mg/ml). Given that hnRNPC does not shuttle between the nucleus and cytoplasm, we also co-stained two FTLD sections with cytoplasmic hnRNPC for anti-Lamin B1 (Abcam, ab16048, 1:500) (Supplementary Fig. 3). Slides were imaged at 100× magnification using the Leica DM6000 microscope. To further assess whether the nuclear loss of hnRNPDL, hnRNPAB, hnRNPC, hnRNPA0 and hnRNPA1 occurred in concert, stripping buffer (LI-COR, Newplot IR) was applied to these sections for 15 min following microwave antigen retrieval (0.01 M citrate buffer, pH 6.0). Sections were then incubated with a primary hnRNP antibody overnight, visualized with AlexaFluor 488-labelled anti-mouse IgG (Thermofisher 1:200) or Alexa 568-labelled anti-rabbit (Thermofisher 1:200) as described above and scanned using the Olympus VS-200 Slide Scanner at 40× magnification. This method was repeated for each primary antibody. Images were analysed using the Olympus VS200 Desktop 4.1.1 software.

### Statistics

Statistical analysis was performed using SPSS, with a value of *P* < 0.05 taken as significant. Data were assessed for normality of distribution using Kolmogorov–Smirnov tests and demographic variables showing nonparametric distribution (sex) were analysed using chi-squared analysis. All other data demonstrated parametric distribution and was analysed using multivariate analyses of variance with bonferonni *post hoc* tests. Pearson’s correlation analysis was conducted to determine any correlations between age, disease duration, pTDP-43 and nuclear hnRNP.

## Results

### Regional hnRNP

**Figure 1:**
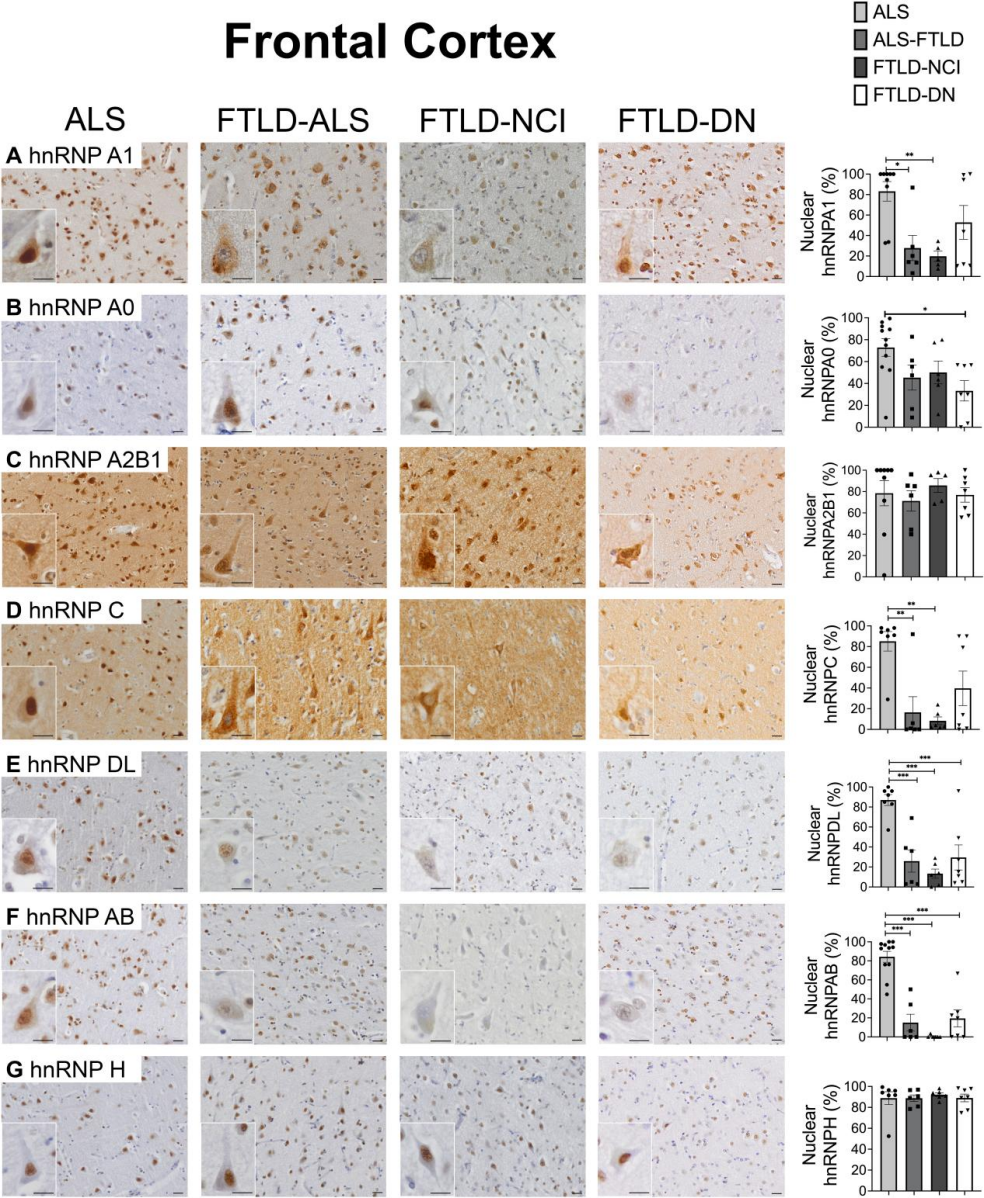
**Nuclear hnRNP.** Normal nuclear hnRNPA1 (**A**), hnRNPA0 (**B**), hnRNPA2B1 (**C**), hnRNPC (**D**), hnRNPDL (**E**), hnRNPAB (**F**) and hnRNPH2 (**G**) were observed in the frontal cortical neurons (Column 1) of ALS cases. In contrast, significant nuclear loss of hnRNPDL (**E**) and hnRNPAB (**F**) was observed in the frontal cortices of FTLD compared to ALS cases (Columns 2–4). Significant nuclear loss of hnRNPA1 (**A**) and hnRNPC (**D**) was observed in the frontal cortices of FTLD-ALS and FTLD-NCI compared to ALS cases. Bonferonni test *P < 0.05, **P < 0.01, *** *P* ≤ 0.001. Scale bars 20 μm.

Nuclear localisation of hnRNPA/B (A1, A0, A2B1), hnRNPC, hnRNPD (DL, AB) and hnRNPH2 was observed in the motor cortical neurons and Betz cells of ALS cases (Fig. 1). In contrast, nuclear depletion of hnRNPA1, hnRNPC, hnRNPDL and hnRNPAB was observed in the frontal cortex of FTLD cases, with this accompanied by cytoplasmic immunoreactivity of hnRNPA1 and hnRNPC (Fig. 1).

### Quantitative analyses

Quantitative analysis revealed no significant difference in nuclear hnRNPA1, A0, A2B1, C, DL, AB or H2 in the motor cortical neurons or Betz cells of ALS compared to FTLD (*P* > 0.1) (Supplementary Fig. 1). In contrast, a significantly greater loss of nuclear hnRNPD (DL and AB) was identified in FTLD-NCI, FTLD-DN and FTLD-ALS compared to ALS cases (*F*(3,26) > 12.3, *P* < 0.001) (Fig. 1). A significantly greater loss of nuclear hnRNPA1 and hnRNPC was also observed in FTLD-ALS and FTLD-NCI compared to ALS cases (*F*(3,26) > 5.8, *P* < 0.005). Nuclear hnRNPA0 was significantly reduced in FTLD-DN compared to ALS cases (*P* < 0.05) but no group differences in hnRNPA2B1 or hnRNPH2 were identified (*P* > 0.1) (Fig. 1). As expected, ALS cases demonstrated rare and significantly less cytoplasmic pTDP-43 inclusions in the frontal cortex but not motor cortex compared to all other FTLD groups (frontal cortex mean ± SEM: 0.003 ± 0.002 in ALS, 0.14 ± 0.05 in FTLD-ALS, 0.15 ± 0.07 in FTLD-NCI, 0.19 ± 0.03 in FTLD-DN; *F*(3, 24) = 8.24, *P* < 0.001; motor cortex mean ± SEM: 0.06 ± 0.01 in ALS, 0.09 ± 0.04 in FTLD-ALS, 0.11 ± 0.02 in FTLD-NCI, 0.11 ± 0.03 in FTLD-DN; *F*(3,22) = 1.42, *P* > 0.2) (Supplementary Fig. 4). As expected, a significantly greater burden of dystrophic neurites was identified in the frontal and motor cortices of FTLD-DN cases compared to all other groups (frontal cortex mean ± SEM: 0.6 ± 0.12 in FTLD-DN, 0.14 ± 0.06 in FTLD-ALS: 0.13 ± 0.08 in FTLD-NCI, 0.009 ± 0.006 in ALS; *F*(3,24) = 16.44, *P* ≤ 0.001; motor cortex mean ± SEM: 0.64 ± 0.12 in FTLD-DN, 0.17 ± 0.06 in FTLD-ALS: 0.05 ± 0.04 in FTLD-NCI, 0.04 ± 0.02 in ALS; *F*(3,22) = 22.4, *P* ≤ 0.001) (Supplementary Fig. 4).

### Correlation analysis

Significant associations were identified between the proportion of neurons with nuclear hnRNPDL, hnRNPAB, hnRNPC, hnRNPA0 and hnRNPA1 in the motor and frontal cortices, with strong relationships (*r* ≥ 0.8) observed between the nuclear hnRNPC and hnRNPA1 (Table 1).

**Table 1:** Correlation matrix displaying correlation coefficients (*r*) between hnRNPs in the frontal and motor cortices of ALS and FTLD cases

	hnRNPA/B			hnRNPC	hnRNPD		hnRNPH
	Nuclear hnRNP A1	Nuclear hnRNP A2B1	Nuclear hnRNP A0	Nuclear hnRNP C	Nuclear hnRNP DL	Nuclear hnRNP AB	Nuclear hnRNP H2
Nuclear hnRNP A1	**1**	0.32*	0.36**	**0**.84**	0.62**	0.69**	0.06
Nuclear hnRNPA2B1	0.32*	**1**	0.35*	0.47**	0.44**	0.27	0.34*
Nuclear hnRNPA0	0.36**	0.35*	**1**	0.29*	0.62**	0.49**	0.24
Nuclear hnRNP C	**0**.84**	0.47**	0.29*	**1**	0.59**	**0**.88**	0.06
Nuclear hnRNP DL	0.62**	0.44**	0.62**	0.59**	**1**	0.78**	0.08
Nuclear hnRNP AB	0.69**	0.27	0.49**	**0**.88**	0.78**	**1**	0.05
Nuclear hnRNP H2	0.06	0.34*	0.24	0.06	0.08	0.05	**1**

Correlation coefficients (*r*) >0.8 are presented in bold.

^*^P < 0.05.

^**^P < 0.005 (Pearson’s correlation *n* = 59).

**Figure 2:**
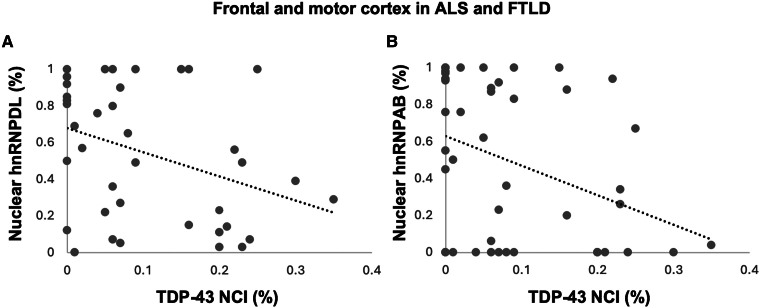
**Correlation analysis.** Increasing pTDP-43 NCI demonstrated a significant negative relationship with nuclear hnRNPDL in the frontal and motor cortices of ALS and FTLD cases (Pearson’s correlation *r* = −0.36, *P* = 0.025, *n* = 39; *y* = −1.32*x* + 0.68) and hnRNPAB (*r* = −0.37, *P* = 0.012; *n* = 47, *y* = −1.6*x* + 0.63) (**A**, **B**).

A significant correlation was also observed between decreasing nuclear hnRNPD (DL and AB) with increasing cytoplasmic pTDP-43 (*ρ* ≥ −0.35, *P* ≤ 0.025) (Fig. 2A and B). No significant associations were identified between pTDP-43 with other hnRNPs.

### Immunofluorescence assessment of hnRNPs

**Figure 3:**
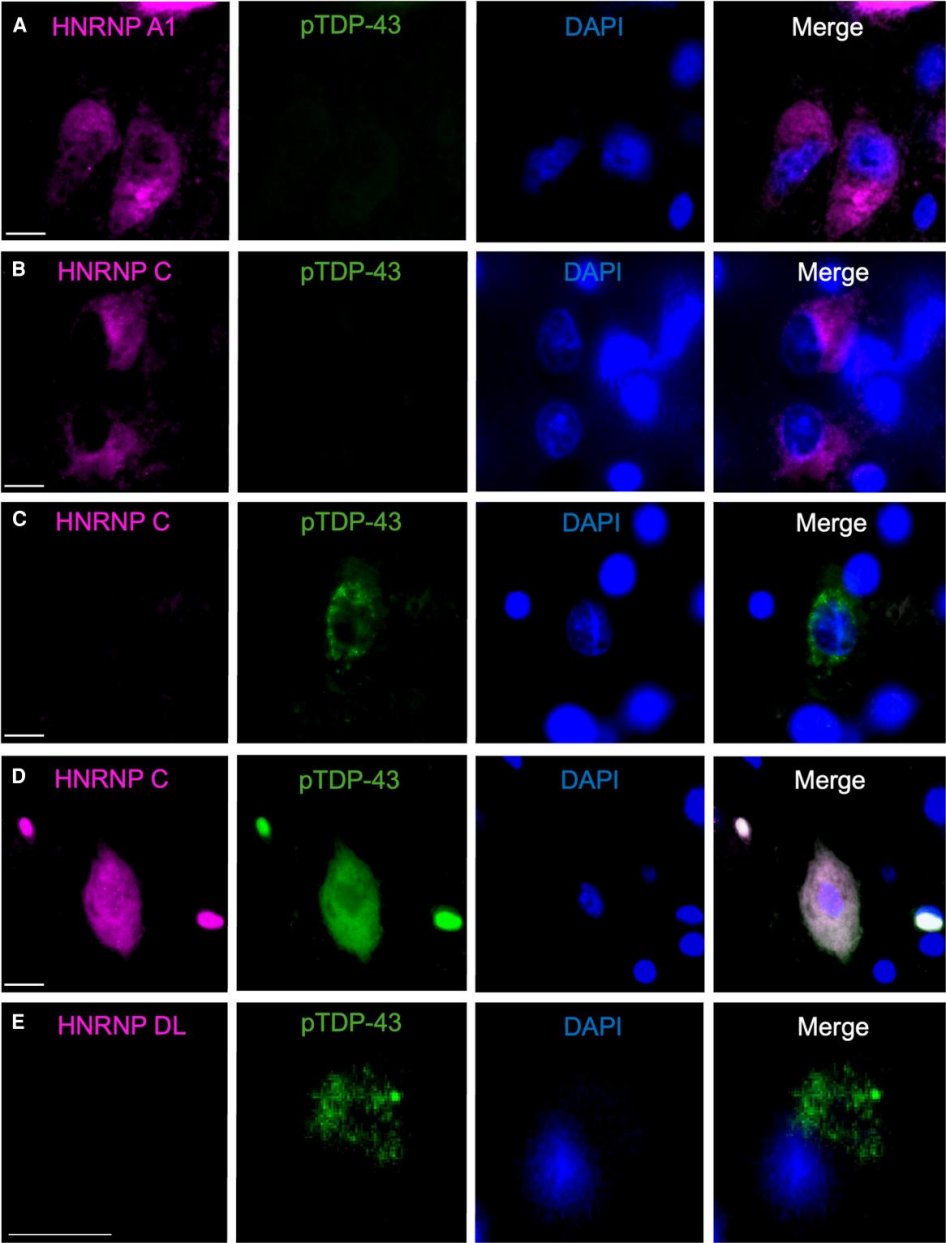
**Co-localisation of hnRNPA1, hnRNPC and hnRNPDL with pTDP-43.** Double-labelled immunofluorescence demonstrated no co-occurrence between cytoplasmic hnRNPA1 and pTDP-43 in FTLD (**A**). Similarly, cytoplasmic hnRNPC and pTDP-43 were predominantly observed in separate neurons in FTLD (**B**, **C**) and only rarely in the same neurons in FTLD (**D**). Nuclear hnRNPDL was not observed in neurons with cytoplasmic pTDP-43 (**E**). Scale bar 10 μm.

**Figure 4:**
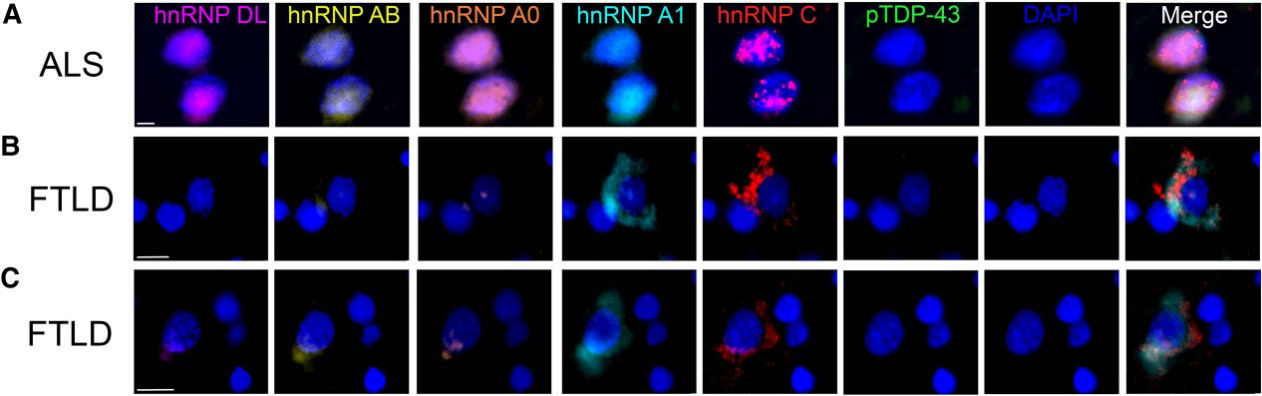
**Co-localisation of hnRNP and pTDP-43.** Normal nuclear localisation of hnRNPDL, hnRNPAB, hnRNPA0, hnRNPA1, hnRNPC in the motor cortex of ALS cases (**A**). In contrast, nuclear depletion of hnRNPDL, hnRNPAB, hnRNP A0, hnRNPA1 and hnRNPC was observed in the frontal cortex of FTLD cases, with cytoplasmic co-occurrence between hnRNPA1 and hnRNPC but not with pTDP-43 observed in the same cells (**B**, **C**). Scale bar 8 μm.

Double-labelled immunofluorescence revealed no co-occurrence between cytoplasmic hnRNPA1 and pTDP-43 cytoplasmic inclusions (Fig. 3A). Similarly, cytoplasmic hnRNPC was predominantly identified in neurons without cytoplasmic pTDP-43 (Fig. 3B) and vice versa (Fig. 3C), with only rare neurons demonstrating cytoplasmic localisation of both hnRNPC and pTDP-43 (Fig. 3D). Multi-labelled fluorescence analysis confirmed normal nuclear co-occurrence of hnRNPDL, hnRNPAB, hnRNP A0, hnRNPA1 and hnRNPC in the motor cortex of ALS cases (Fig. 4A). In contrast, nuclear loss of hnRNPDL, hnRNPAB, hnRNP A0, hnRNPA1 and hnRNPC were concurrently observed in neurons of the frontal cortex in FTLD cases, with co-occurrence of cytoplasmic hnRNPC and hnRNPA1 observed (Fig. 4B and C).

## Discussion

This comparative analysis of seven hnRNPs across the ALS-FTLD continuum revealed significantly greater loss of nuclear hnRNPD (DL, AB), hnRNPC and hnRNPA1 in the frontal cortex of cases with sporadic FTLD compared to ALS. Despite the greater burden of dystrophic neurites, no significant difference was found in nuclear hnRNPs in FTLD-DN compared to FTLD-NCI cases. This is consistent with the similar cytoplasmic pTDP-43 burden identified at the end of the significantly longer disease duration in FTLD-DN cases with that in FTLD-NCI cases. Cytoplasmic co-occurrence of hnRNPA1 and hnRNPC was observed in the frontal cortex of FTLD cases but pTDP-43 did not co-localize with cytoplasmic hnRNPA1 and was only rarely observed in neurons with cytoplasmic hnRNPC. Although the motor cortex is an early region implicated in ALS that is only affected later in FTLD, no significant differences in nuclear hnRNPs were identified in the motor cortical neurons or Betz cells between ALS and FTLD cases. Interestingly, nuclear loss of hnRNPD (DL, AB) was found to associate with increasing cytoplasmic pTDP-43 as well as with decreasing nuclear hnRNPA1, A0 and C to suggest the involvement of this understudied subfamily in the pathogenesis of TDP-43 proteinopathies.

HnRNPD is preferentially localized to nuclear speckles where it regulates multiple alternative splicing events and has been shown to be a strong functional modulator of TDP-43.^5,6^ Nuclear depletion and/or mutations in hnRNPD has been linked to limb-girdle muscular dystrophy, underscoring the importance of this normally nuclear hnRNP.^22^ Silencing of TDP-43 in cultured human brain pericytes has been found to cause alternative splicing of hnRNPD.^23^ The significant relationship identified between increasing nuclear hnRNPD loss and cytoplasmic pTDP-43 here further corroborates a regulatory role between these two hnRNPs.

hnRNP C is a non-shuttling protein which is normally expressed in the nucleus of neurons in human brain tissue^24^ and has been identified, albeit rarely, in neuronal cytoplasmic inclusions (NCI) in the hippocampus of neuronal intermediate filament inclusion disease.^18^ Similar to hnRNPD, loss of hnRNPC has also been shown to exacerbate TDP-43 induced dysfunction in experimental models^5^ and its independent depletion causes a considerably greater number of mis-splicing events compared to that seen with TDP-43 loss.^25^ Despite this, histological assessments of hnRNPC in TDP-43 proteinopathies is scarce. We demonstrate here significant nuclear loss of hnRNPC in the frontal cortex of FTLD cases characterized by cytoplasmic inclusions but only rare co-occurrence with pTDP-43 aggregates, suggesting independent pathways in FTLD. Importantly, hnRNPC and hnRNPD levels can be detected in plasma samples and has been found to be significantly reduced in patients with bulbar compared to limb-onset ALS,^26^ highlighting the need for further investigation into the potential of these hnRNPs to serve as proximal markers of TDP-43 dysfunction and/or disease subtypes, particularly since hnRNPC is already recognized as a promising biomarker in various cancers.^27^

Studies in experimental models have found increased hnRNPA1 transcript and cytoplasmic hnRNPA1 protein levels in TDP-43 depleted cells.^28^ Consistent with this, and previous findings of cytoplasmic hnRNPA1 in post-mortem ALS cases,^29,30^ we demonstrate significant nuclear depletion accompanied by cytoplasmic immunoreactivity of hnRNPA1 in the frontal cortex, which is the predilection site of TDP-43 dysfunction in FTLD and FTLD-ALS cases. Consistent with that seen in the lower motor neurons,^31^ nuclear loss of hnRNPA1 was not observed in the motor cortex of ALS cases here. Importantly, various hnRNPA1-targeting compounds have been developed and are currently being trialled in cancers.^7^ However, in contrast to the increased nuclear hnRNPA1 identified in most cancers^32,33^ the present findings of reduced nuclear hnRNPA1 in FTLD suggest the need for further studies to determine whether these compounds seeking to mediate nuclear loss or retain cytoplasmic hnRNPA1^7,34^ will be appropriate for TDP-43 proteinopathies. Nevertheless, the potential druggability of hnRNPA1 coupled with its association with hnRNPC and hnRNPD here provides an important avenue for future studies to determine whether hnRNPs and consequently TDP-43, can be modulated via hnRNPA1 in ALS and FTLD.

No significant difference in nuclear hnRNPs were observed in the motor cortex between ALS and FTD, despite this being a predilection region of TDP-43 dysfunction in ALS. Although nuclear depletion of TDP-43 is observed in both motor cortical neurons and Betz cells in ALS, cytoplasmic aggregates of TDP-43 occur in motor cortical neurons and rarely in Betz cells.^20,35^ Despite this, both neuronal populations demonstrated no significant difference in nuclear hnRNPs between ALS and FTLD cases here, consistent with the similar TDP-43 burden in this region across ALS and FTLD cases^9^ raising the question as to whether the nuclear preservation of these hnRNPs in this predilection region in ALS contributes to the relatively milder pTDP-43 aggregation.^9^ However, age-matched controls were not available and our comparison of the proportion of neurons with normal nuclear hnRNPs revealed some nuclear loss of hnRNPD and hnRNPC of ALS cases which although not significant compared to FTLD-DN, does not exclude the involvement of these hnRNPs in this milder TDP-43 proteinopathy disease, highlighting an important future direction alongside the study of these hnRNPs in the lower motor neurons. The immunogen sequences of the antibodies used to detect hnRNPDL, hnRNPAB, hnRNPC and hnRNPA0 demonstrated a low likelihood of cross-reactivity.^36^ Further to this, the hnRNPDL antibody employed here was previously used to assess HeLa hnRNPDL knockout cell lines and confirm nuclear localisation in cells transiently transfected with hnRNPDL.^37^ However, the immunogen sequences of the antibodies employed to detect hnRNPA1, A2B1 and H2 proteins here are likely to cross-react within their subfamilies and this is a methodological limitation that warrants consideration. Importantly, in contrast to hnRNPD and C, these three hnRNPs have been previously studied in tissue from patients with ALS and FTD and consistent with our current findings in the motor cortex, normal nuclear localisation of hnRNPA2B1 and H2 but not A1 were also identified in the lower motor neurons of ALS.^29-31,38^ Future studies in experimental models will be needed to determine the mechanistic involvement and interaction of these nuclear hnRNPs in ALS and FTLD. As with most quantitative pathological studies in human tissue, group sizes were relatively small and although future replication in a large sample size will further strengthen these findings, the striking and consistent nuclear hnRNP loss observed in FTLD cases here provides strong support that our results are representative of this cohort.

In summary, the present study demonstrates significant nuclear loss of hnRNPD, hnRNPC and hnRNPA1 in the frontal cortex of FTLD compared to ALS cases, which is consistent with the greater burden of pathological TDP-43 in this region in sporadic FTLD compared to ALS. In contrast, no significant difference in these hnRNPs was observed in the motor cortical neurons or Betz cells in ALS compared to FTLD cases. These findings implicate these hnRNPs and suggest the involvement of widespread mis-splicing events in the pathogenesis of sporadic FTLD and highlight the need for further investigations to determine whether they contribute to the distinct pathomechanisms increasingly recognized across the ALS-FTLD continuum, as well as to assess their potential as targets and/or proximal markers of TDP-43 dysfunction.

## Supplementary Material

fcae352_Supplementary_Data

## Data Availability

Anonymized datasets generated during this study are available from the corresponding author on reasonable request.
