# Supplementary material for: Heterogeneous nuclear ribonucleoprotein D — an understudied subfamily affected in sporadic TDP-43 proteinopathies
Source: Brain Commun. 2024 Nov 1;6(6):fcae352. doi: 10.1093/braincomms/fcae352 (PMC11635367; doi:10.1093/braincomms/fcae352)
Supplement: fcae352_Supplementary_Data [file fcae352_Supplementary_Data.pdf]

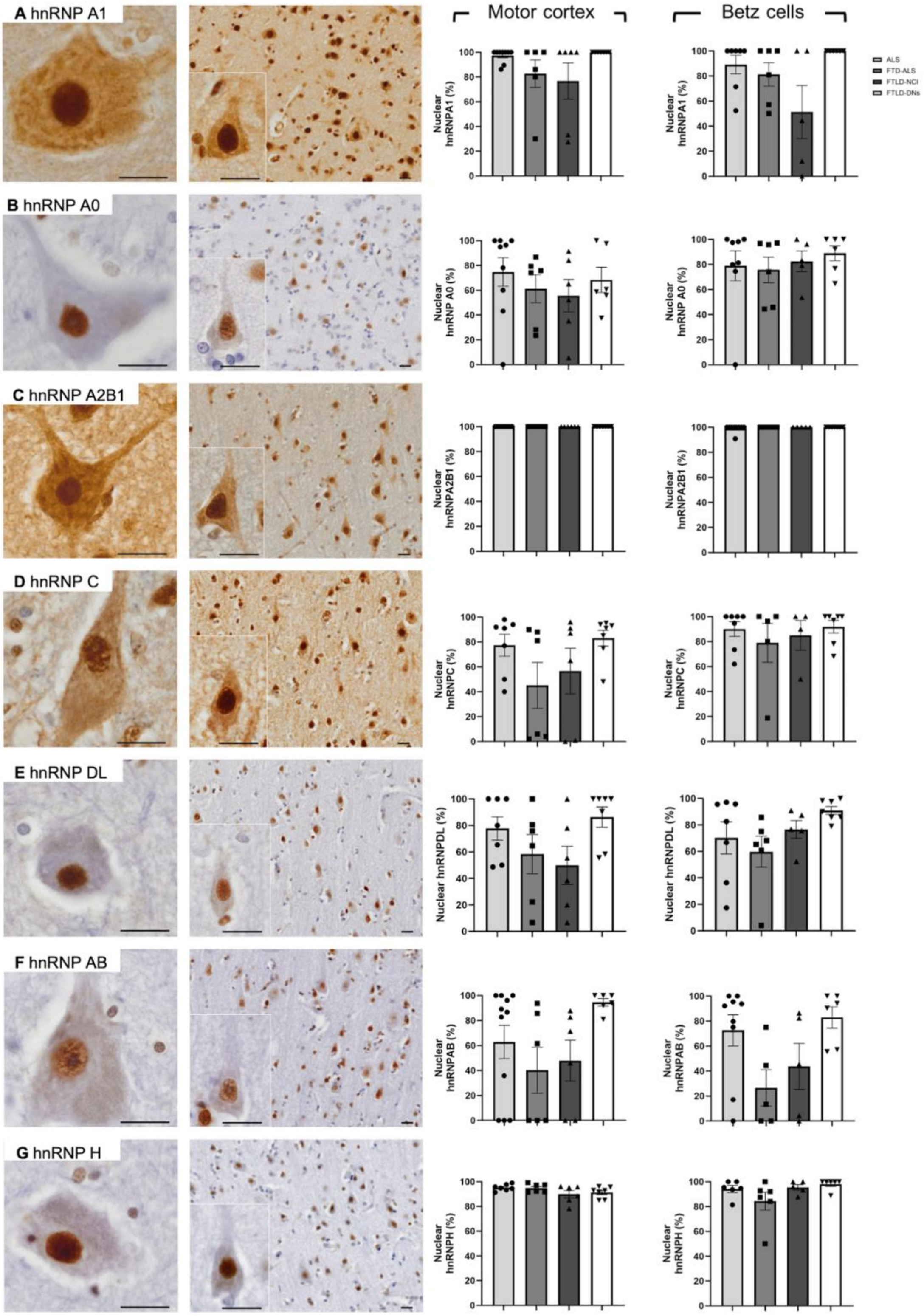

Supplementary Figure 1. Normal nuclear hnRNPA1, hnRNPA0, hnRNPA2B1, hnRNPC, hnRNPDL, hnRNPAB and hnRNPH2 were observed in the Betz cells and motor cortical neurons in the motor cortex with no significant difference in the proportion of neurons with normal nuclear hnRNPs identified between ALS and FTLD cases (ANOVA $F(3,24)>1.76$, $p>0.1$). Scale bars 20 μm.

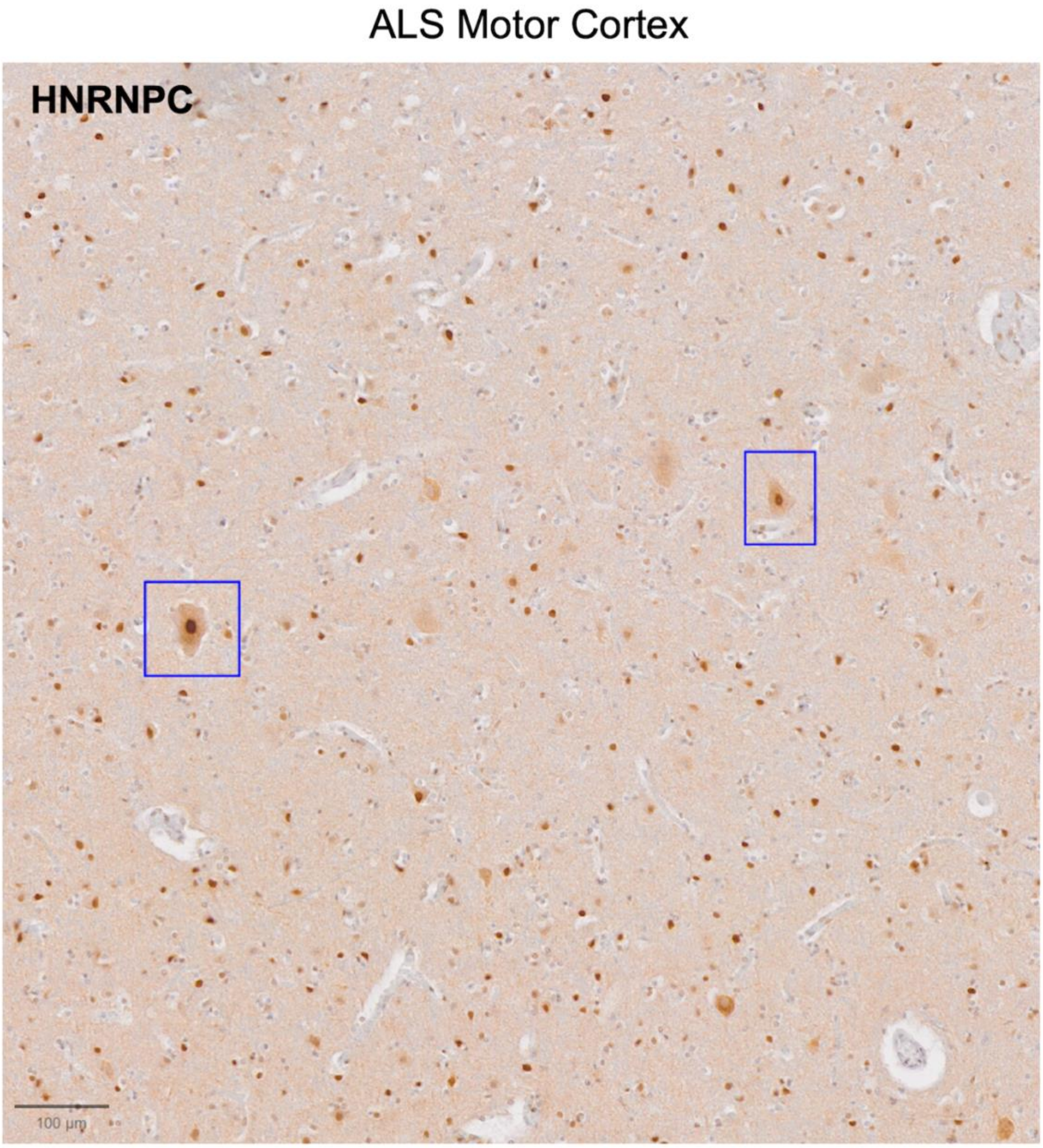

Supplementary Figure 2. Betz cells were identified in the motor cortex by their large soma at lower magnification (10x) across the layer. Example of Betz cells with hnRNPC in the ALS motor cortex is shown. Scale bar 100 µm.

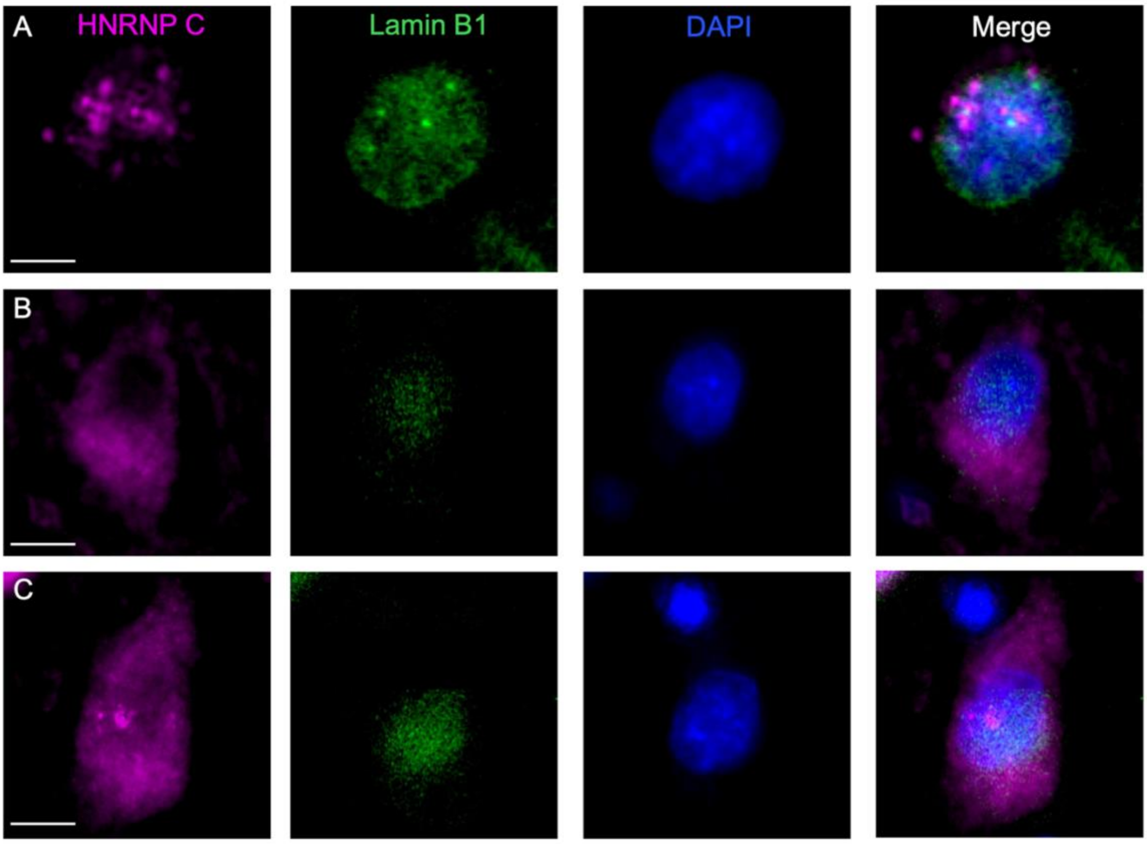

Supplementary Figure 3. Double-labelled immunofluorescence demonstrated Lamin-B1 in the nucleus of a neuron with normal hnRNPC from an ALS case (A) and mild loss of Lamin B1 in the nucleus of neurons with cytoplasmic hnRNPC from an FTLD case (B, C). Scale bar 5 μm.

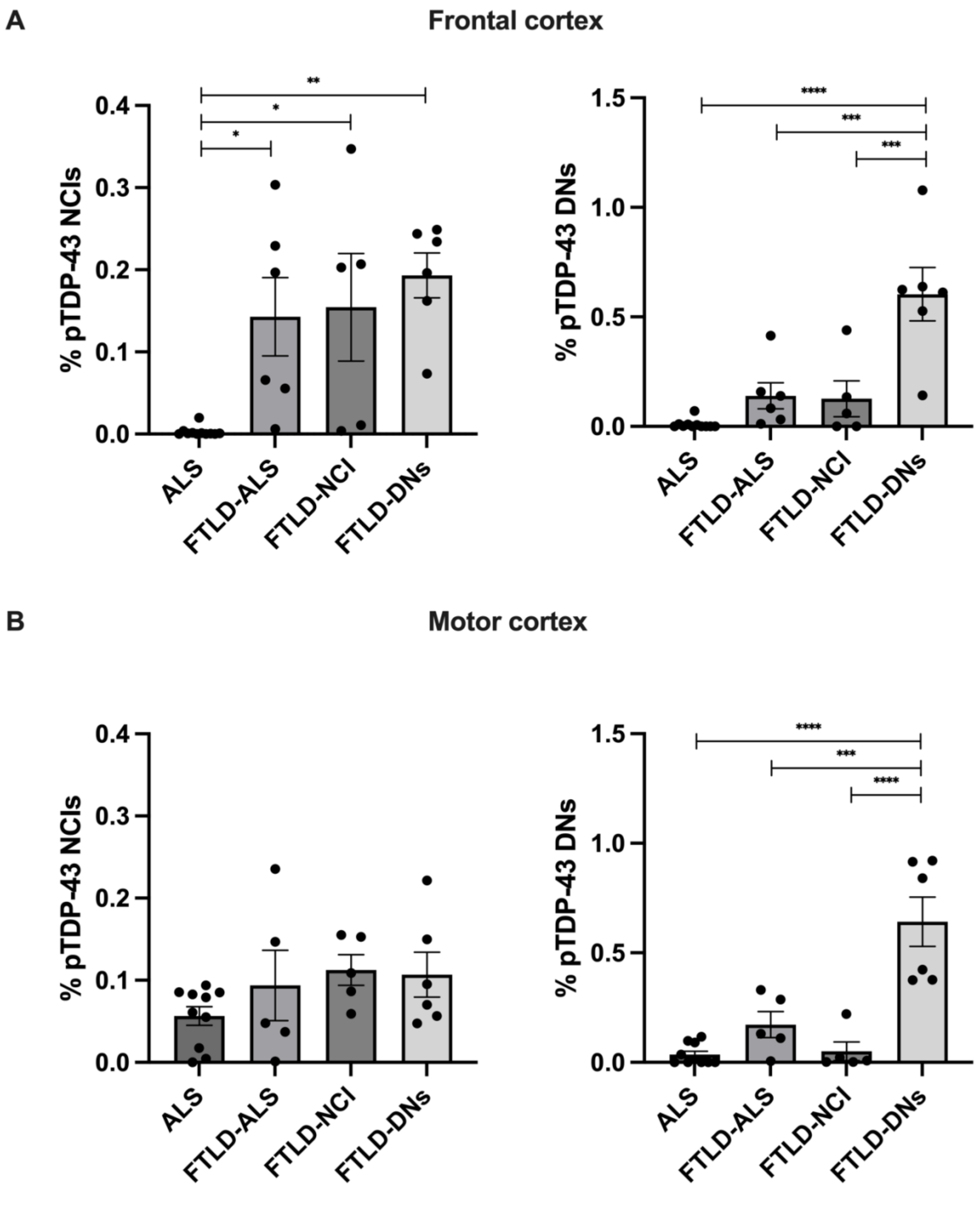

Supplementary Figure 4. Percentage burden of pTDP-43 neuronal cytoplasmic inclusions (NCIs) and dystrophic neurites (DNs) in the frontal cortex (A) and motor cortex (B) of ALS, FTLD-ALS, FTLD-NCI and FTLD-DN cases. One-way ANOVA followed by Bonferonni test *p<0.05, ***p≤0.001.
